# Single-cell dynamic RNA and glycosylation sequencing reveals the mechanism underlying the differentiation of pluripotent stem cells into hematopoietic stem cells

**DOI:** 10.1007/s13577-025-01234-7

**Published:** 2025-05-27

**Authors:** Wanyi Feng, Sheng Zeng, Donghui Liu, Wei Gong, Junjie Hu, Weihua Xu, Zhichao Ma, Shengmiao Fu, Xinping Chen

**Affiliations:** 1 School of Life and Health Sciences, Hainan University, 58th People’s Avenue, Haikou, Hainan China; 2 Affiliated Cancer Hospital of Hainan Medical University, 4th Changbin West Street, Haikou, 570100 Hainan China; 3 Susheng Biotech (Hainan) Co., Ltd, 1st Medicine Valley Road, Haikou, Hainan China; 4 Academician Innovation Platform of Hainan Province, Haikou, Hainan China

**Keywords:** Induced pluripotent stem cell, Hematopoietic stem/progenitor cell, Single-cell glycosylation sequencing, Single-cell dynamic RNA sequencing, Hematopoiesis differentiation

## Abstract

Studying the mechanism of hematopoietic stem cells’ generation from induced pluripotent stem cells in vitro can be useful for understanding embryonic hematopoiesis, as well as for the application of related cell therapy. This study aimed to delineate the process of the differentiation of induced pluripotent stem cells into hematopoietic stem cells’ models and provide a theoretical basis and clinical value for the production of hematopoietic stem cells in vitro. We analyzed the differentiation model by single-cell dynamic transcriptome and glycosylation sequencing, which was divided into three differentiation stages based on the new‐to‐total RNA ratio and glycosylation level. Two differentiation fates were found in the pseudo-time, including hematopoietic development and other tissue development. Precursor hematopoietic cells with a high glycosylation level greatly expressed hematopoietic regulation and vascular endothelial genes, suggesting that glycosylation is associated with angiogenesis and hematopoietic regulation. The multiple differentiation events in the in vitro model are similar to those in hematopoietic development in vivo, including yolk sac hematopoiesis, cellular communication between non-potential hematopoietic subsets and potential hematopoietic subsets, gene expression, and temporal deviations in hematopoietic fate. Our study has revealed the similar hematopoiesis process in the differentiation model via single-cell dynamic RNA and glycosylation sequencing, which provides an important theoretical basis for the study of hematopoietic stem cell development.

**Supplementary Information:**

The online version contains supplementary material available at 10.1007/s13577-025-01234-7.

## Introduction

The hematologic system is one of the earliest systems to form during embryonic development in mammals [1]. Hematopoietic cells are derived from mesoderm cells (MCs). This process can be achieved in three ways: primitive hematopoiesis, pro-definitive hematopoiesis, and definitive hematopoiesis [2, 3]. The same hematopoietic cells in different stages may have different functions; for example, primitive macrophages specialize as microglia involved in brain development, while hematopoietic stem cell (HSC)-derived macrophages are involved in immune regulation [4, 5]. Primitive hematopoiesis and pro-definitive hematopoiesis occur outside the embryo in the yolk sac, and various hematopoietic cells are generated through HSC-independent differentiation [2, 3, 6]. Primitive hematopoiesis supports embryo development and helps maintain oxygenation, while pro-definitive hematopoiesis establishes the initial hematopoietic system [7] and sustains hematopoiesis before birth. Definitive hematopoiesis occurs ventral to the dorsal aorta in the aorta-gonad-mesonephros (AGM) region of the embryo [6, 8] and gives rise to HSCs, which maintain lifelong hematopoietic functions. HSCs are generated from a group of specialized endothelial cells known as hemogenic endothelial cells (HECs), which are located within the vascular endothelium [9]. These HECs form intra-aortic hematopoietic clusters (IAHCs) along the blood vessel wall and undergo an endothelial-to-hematopoietic transition (EHT) to produce HSCs [10].

The differentiation of hiPSCs into hematopoietic stem/progenitor cells (HSPCs) in vitro is a valuable model for studying embryonic hematopoiesis. It not only provides insights into the mechanisms underlying hematopoiesis but also provides a method to test potential clinical applications of hematopoietic cells. Regarding this model, the generation of HSPCs from hiPSCs also follows a phased process. It has been reported that HSPCs from this model have successfully differentiated into functional terminal hematopoietic cells, including erythrocytes and eosinophils [11, 12]. Generally, iPSCs are stimulated by factors such as activin A and BMP4 to differentiate into MCs that have strong potential for vascular differentiation. These MCs are later stimulated by VEGF and bFGF to generate vascular endothelial cells (VEC) [11, 13, 14]. Then, HECs (a type of VEC) that have both endothelial and hematopoietic properties then produce HSCs through the EHT [15]. Several studies have identified several signaling pathways and transcription factors involved in regulating the formation of HSPCs, including factors, such as RUNX1, GATA2, BMP4, and TAL1, and pathways such as TGFβ [11, 13, 14, 16].

The degree of cell glycosylation has significant biological implications; for example, high-level glycosylation is associated with functional proteins and influences the activity and efficiency of cell signal transduction pathways. Glycosylation plays a regulatory role in EHT, as shown by microRNA-223-mediated N-glycan biosynthesis in endothelial cells, which regulates hematopoietic development [17]. O-GlcNAc regulates the balance of early development of skeletal and hematopoietic systems through RUNX2 and C/EBPβ and plays an important role in the establishment of bone marrow niche for hematopoiesis by the intracellular glycosylation process of specific proteins [18]. However, the precise relationship between glycans and RNA in the context of hematopoietic development is not clear.

Single-cell sequencing is a valuable technique for studying cellular heterogeneity and has been widely used to interpret cell development events. For in vitro hematopoiesis, the dynamics and heterogeneity of HSPC populations make it challenging to investigate the molecular mechanisms associated with differentiation. In this study, we analyzed the transcriptome profile and glycosylation levels using a combination of single-cell RNA sequencing (scRNA-seq), single-cell dynamic RNA sequencing (DynaSCOPE), and single-cell glycosylation sequencing (ProMoSCOPE) technologies, which have helped us to understand the interactions between cellular and molecular mechanisms during hematopoiesis in vitro, and the effect of glycosylation levels on hematopoietic regulation. This study provides a theoretical basis for the generation of functional HSPCs using iPSCs.

## Methods

### Human-induced pluripotent stem cell culture and differentiation

The Clone10 hiPSC line was used in this study. The Clone10 hiPSC line was originally generated from MRC5 cell line (a commercial human fibroblast cell line, Fuyu Biotechnology Co., Ltd Catalog #JY198) using CytoTune™ -iPS 2.0 Sendai Reprogramming Kit (Thermo Fisher Catalog #A34546) and qualified in our lab (data not shown). For hiPSCs’ culture, each well was coated with 1 mL of Matrigel (Corning Catalog #354277) at least 1 h before plating, and the cells were passaged with 1 mL of ReLeSR (Stem Cell Catalog #100–0483). The hiPSCs were cultured in mTeSR™ plus medium (Stem Cell Catalog #100–0276) containing 1% penicillin/streptomycin (Gibco Catalog #15070–063). The cells were cultured in an air-humidified incubator at 37 ℃, supplemented with 5% CO_2_ until 60–80% confluency was achieved. The medium was changed every day.

Undifferentiated hiPSCs were dissociated into a single-cell solution using Accutase (Gibco Catalog #A6964) and were seeded on Matrigel (Corning Catalog #354277)-coated 6-well plates at a density of 3–6 × 10^4^ cells/dish in mTeSR™ plus medium for 1–2 days before hematopoietic differentiation induction. For hematopoietic differentiation, serum-free Iscove's modified Dulbecco's medium (IMDM, Gibco Catalog #12440–053) supplemented with 2% B27 (without vitamin A, Gibco Catalog #12587–010), 50 μg/mL L-ascorbic acid (Sigma-Aldrich Catalog #A4403), 0.5% Penicillin/Streptomycin, and 1% GlutaMAX-I (Gibco Catalog #2277243) was used (termed as basic differentiation medium, BDM). On day 0 of differentiation, 20 ng/mL Activin A (MCE Catalog #HY-P70311), 20 ng/mL bone morphogenetic protein 4 (BMP4, MCE Catalog #HY-P7007), and 5 μM CHIR-99021 (MCE Catalog #HY-10182) were added to the BDM. From day 2 to day 6, 50 ng/mL human vascular endothelial growth factor (VEGF, MCE Catalog #HY-P70458), 50 ng/mL human basic fibroblast growth factor (bFGF, MCE Catalog #HY-P7331), 5 ng/mL BMP4, and 10 μM SB-431542 (MCE Catalog #HY-10431) were added to the BDM. From day 6 to day 10, 5 ng/mL BMP4, 10 ng/mL VEGF, 2 U/mL erythropoietin (EPO, MCE Catalog #HY-P7164), 20 ng/mL recombinant human stem cell factor (SCF, MCE Catalog #HY-P70757G), 15 μM NAC (Sigma-Aldrich Catalog #A7250-5G), and 1 μM minocycline hydrochloride (Selleck Catalog #S4226) were added to the BDM. Half of the medium was replaced daily beginning on day 6.

### Flow cytometry analysis and cell sorting

The cells from days 0, 2, 4, 6, 8, and 10 were collected and dissociated into single cells and washed with 1% bovine serum albumin (BSA, Sigma-Aldrich Catalog #V900933) in phosphate-buffered saline (PBS, Gibco Catalog #AM9624) (BSA/PBS) buffer, and then, 5 × 10^5^ cells per 100 μL were placed into a single tube. The cell suspension in BSA/PBS buffer was stained with desired antibodies for 30 min at room temperature under gentle shaking. The cell suspensions were analyzed using CytoFLEX LX (Beckman Coulter), and the flow cytometry data were recorded using the CytExpert software (Beckman Coulter, v2.4.0.28). The antibodies used in this study are described in Supplementary Table 1. The cells were sorted with BD Melody (BD) and collected in a differentiation medium.

### Colony-forming assay

The cells were collected on day 10, and CD34^+^CD43^+^ HSPCs were subjected to FACS. A total of 3 × 10^3^ cells/dish were resuspended in 100 μL of IMDM supplemented with 2% FBS, and 1 mL of Methocult H4435 (Stem Cell Catalog #H4435) was added and mixed thoroughly. The cells were incubated at 37 °C in 5% CO_2_ for 13–16 days. The colony-forming units (CFUs) were classified and counted according to their morphological characteristics.

### Metabolic labeling in vitro

Labeling experiments were conducted following the protocol provided with the DynaSCOPE® Single Cell Dynamic RNA Library Kits (Singleron Biotechnologies Catalog #1251011). Briefly, the labeling culture medium was prepared according to the instruction, and then, the labeling reagent was added to the medium at a ratio of 1:100. The prepared single cells were transferred to the labeling culture medium, and kept in the dark for 2 h. After incubation, the supernatant was discarded, and the cell pellet was resuspended in an appropriate volume of PBS.

### Glycosylation labeling

The samples underwent glycosylation labeling with the ProMoSCOPE™ Single Cell Glycosylation Detection Kit (Singleron Biotechnologies Catalog #1251012) following the manufacturer's instructions. Briefly, the cells were prepared, and the glycosylation sites on the cell surface were labeled with fucose residues that had a tag containing a PCR handle, a known sequence, and a poly A sequence. After washing thrice to remove excess labels, the cells were resuspended with PBS and adjusted to an appropriate concentration. Subsequent operations were performed according to the manual.

### Reverse transcription, amplification, and library construction

Single-cell suspensions (2 × 10^5^ cells/mL) formed with PBS (HyClone Catalog #SH30256.01) were loaded onto a microwell chip using the Singleron Matrix® Single Cell Processing System. Barcoding Beads were subsequently collected from the microwell chip, followed by reverse transcription of the mRNA captured by the Barcoding Beads, to synthesize cDNA. The cDNA synthesized was used for PCR amplification. The amplified cDNA was fragmented and ligated with sequencing adapters. The scRNA-seq libraries were constructed according to the protocol provided with the GEXSCOPE® Single Cell RNA Library system [19]. Individual libraries were diluted to 4 nM, pooled, and sequenced on Illumina Novaseq 6000 with 150 bp paired-end reads.

### Preprocessing

Barcodes and UMIs were extracted from R1 reads and corrected. Adapter sequences and poly A tails were trimmed from R2 reads using Cutadapt v3.7.

### Single-cell RNA-seq library analysis

After preprocessing, R2 reads were aligned against the GRCh38 transcriptome using STAR v2.6.1b. Uniquely mapped reads were then assigned to exons with FeatureCounts v2.0.1. Successfully assigned reads with the same cell barcode, UMI, and gene were grouped to generate the gene expression matrix for further analysis.

### Single-cell proMoSCOPE library analysis

The 15 bp tag sequence was extracted from the R2 reads according to the position, and compared with the known glycosylation tag sequence. A tag sequence with a Hamming distance less than 2 was considered a valid tag. The number of UMI tags contained in each cell barcode is counted to generate a single-cell glycosylation UMI expression matrix. Centered log-ratio transformation was used to normalize the glycosylation UMI of each sample. The cells were determined to be glycosylation-low or glycosylation-high via the median value of normalized UMI.

### Quality control, dimension-reduction, and clustering (scanpy)

Quality control, dimensionality reduction, and clustering analysis were performed using Scanpy v1.8.2 under Python v3.9.10. For each sample dataset, the expression matrix was filtered based on the following criteria: (1) cells with a gene count less than 200 or with a top 2% gene count were excluded; (2) cells with a top 2% UMI count were excluded; (3) cells with mitochondrial content greater than 20% were excluded; (4) genes expressed in less than 5 cells were excluded. After applying the filtering criteria, a total of 168,476 cells were retained for downstream analyses. On average, each cell had 2302 genes and 5974 UMIs. The raw count matrix was then normalized by total counts per cell and logarithmically transformed to generate a normalized data matrix. The top 2000 variable genes were selected using the flavor ='seurat'in Scanpy. Principle Component Analysis (PCA) was performed on the scaled variable gene matrix, and the top 20 principle components were used for clustering and dimensional reduction. The batch effect between samples was removed by Harnomy [20]. After batch correction, the cells were separated into 18 clusters using the Louvain algorithm with a resolution parameter set at 1.2. The resulting cell clusters were visualized using Uniform Manifold Approximation and Projection (UMAP). Matrices of new, old, and total transcripts were used in further analysis.

### Differentially expressed genes (DEGs) analysis (seurat)

To identify differentially expressed genes (DEGs), we used the FindMarkers() function based on the Wilcoxon rank sum test with default parameters and selected the genes expressed in more than 10% of the cells in either of the compared groups of cells and with an average log (Fold Change) value greater than 0.25 as DEGs. The adjusted p values were calculated using the Bonferroni correction method. A significance threshold of 0.05 was used to evaluate the statistical significance of the DEG.

### Pathway enrichment analysis

To investigate the potential functions of DEGs between clusters, Gene Ontology (GO) analysis were used with the “clusterProfiler” R package v4.0.0. [21]. This package provides functions to analyze and visualize the enrichment of biological pathways and gene ontology terms associated with the DEGs. Pathways with adjusted p value less than 0.05 were considered as significantly enriched. Selected significant pathways were plotted as bar plots.

### Subtyping of major cell types

To obtain a high-resolution map of HECs and HSPCs from the specific cluster were extracted and reclustered for more detailed analysis following the same procedures described above and by setting the clustering resolution to 0.5.

### Pseudotime trajectory analysis: monocle2

The cell differentiation trajectory of monocyte subtypes was reconstructed using the Monocle2 v2.22.0. [22]. To select the top 2000 highly variable genes, the FindVariableFeatures() function from Seurat v4.1.0 was used. Dimension reduction was performed using the DDRTree() function. The trajectory was then visualized using the plot_cell_trajectory() function in Monocle2. This analysis allows for the visualization and interpretation of the developmental trajectory of monocyte subtypes. BEAM approach in Monocle was used to test whether differences in gene expression are associated with particular branching events on the trajectory.

### RNA velocity

For RNA velocity, BAM files containing total cells, HECs, HSCs, and the reference genome GRCh38 (hg38) were used for analysis with velocyto v0.17.17 [23] and scVelo v0.2.4 in python with default parameters. The RNA velocity results were projected onto the UMAP plot obtained from the Seurat clustering analysis.

### Functional gene module analysis (hotspot)

Hotspot was used to identify functional gene modules that illustrate heterogeneity within cluster 1 and 15 subpopulations [24]. Briefly, we used the ‘danb’ model and selected the top 500 genes with the highest autocorrelation zscore for module identification. Modules were then identified using the create_modules function, with min_gene_threshold = 15 and fdr_threshold = 0.05. Module scores were calculated using the calculate_module_scores function.

### Expression pattern cluster

We used Mfuzz v2.58.0 [25] to identify time-dependent transcriptional programs in D0, D4, and D8 total cells. The samples were grouped according to the disease progression stage. First, the average expression of each gene was calculated for each stage. Next, more than 25% of the genes that were measured to be missing were excluded. Replace the remaining deletion values with the mean expression values of the corresponding genes. Then, the “filter.std(min.std = 0)” and “standardize()” functions were performed for preprocessing according to the tutorial.

### UCell gene set scoring

Gene set scoring was performed using the R package UCell v2.2.0. [26] UCell scores are calculated based on the Mann–Whitney U statistic, which ranks query genes according to their expression levels in individual cells. UCell is a rank-based scoring method, making it suitable for large datasets that contain multiple samples and batches. This approach allows for the assessment of gene set activity across cell types.

### Transcription factor regulatory network analysis (pySCENIC)

The transcription factor network was constructed using the pyscenic v0.11.0. [27] This was done by utilizing the single-cell RNA expression matrix and transcription factors from the AnimalTFDB. First, the GRNBoost2 predicted a regulatory network based on the co-expression patterns of regulators and their target genes. This network represents potential regulatory interactions between transcription factors and their target genes. Next, the CisTarget was applied to exclude indirect targets and search for transcription factor binding motifs. After constructing the regulatory network, the AUCell algorithm was used to quantify the activity of regulons in each cell. To identify cluster-specific transcription factor regulons, the Regulon Specificity Scores (RSS) were calculated. The activity of these cluster-specific transcription factor regulons was visualized using heatmaps.

### Cell–cell interaction analysis: cellPhoneDB

Cell–cell interactions (CCI) between 18 clusters were predicted using the Cellphone DB package version 4.0.0 [28]. This analysis was based on known ligand–receptor pairs, which are molecules involved in intercellular communication. The permutation number for calculating the null distribution was set to 1000, providing a robust estimate of the expected expression levels for each ligand–receptor pair. The individual ligand or receptor expression was thresholded using a cutoff based on the average log gene expression distribution for all genes across each cell type. Predicted interaction pairs with p value < 0.05 and of average log expression > 0.1 were considered as significant and visualized by heatmap_plot and dot_plot in CellphoneDB.

## Results

### Inducing the differentiation of hiPSCs into HSPCs in vitro

**Fig. 1:**
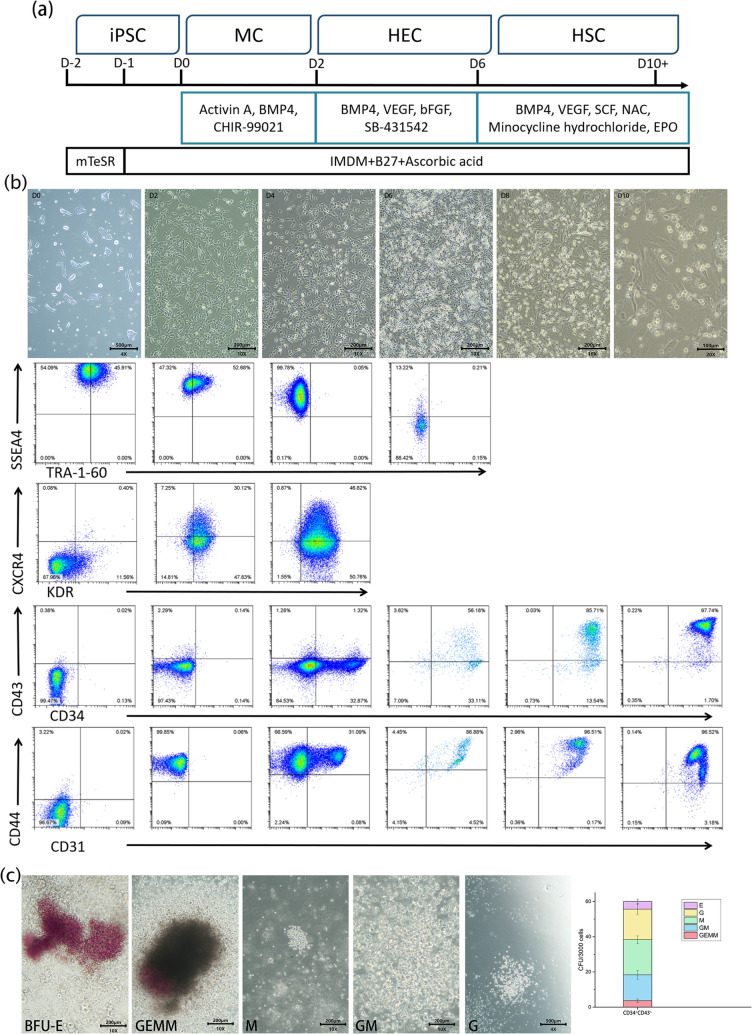
The differentiation of iPSC-to-HSPC in vitro. **a**. The protocol for hematopoietic differentiation of iPSCs. **b**. Changes in cell morphology during differentiation (D0, D2, D4, D6, D8, and D10) and corresponding flow cytometry examination (FCM) of related surface markers. **c**. The images and the number of CFUs collected from CD34 + CD43 + HSPCs on D10 (magnification: 10X). *BFU-E* erythroid cells, *GEMM* granulocytes, erythrocytes, macrophages, and megakaryocytes, *GM* granulocytes and macrophages, *M* macrophages, *G* granulocytes

To study the changes in the transcriptome dynamics and glycosylation level during hematopoietic development in vitro, a protocol to induce gradual differentiation of hiPSCs into HSPCs was formulated [11, 14]. Here, hematopoietic induction was performed using the Clone10 hiPSC cell line (Fig. 1a). Before differentiation, the cells were plated into 6-well plates coated with Matrigel to maintain cell pluripotency. hiPSCs were first treated with BMP4, CHIR-99021, and Activin A for 2 days. This treatment induced the generation of MCs from hiPSCs (labeled with KDR^+^). Then, the three compounds were replaced with VEGF, bFGF, and SB-431542 and cultured for 4 days to induce HECs (labeled with CD34^+^CD31^+^). Finally, EPO, NAC, Minocycline Hydrochloride, and SCF were added for 4 days to induce CD34^+^CD43^+^ HSPCs.

In the in vitro differentiation model of HSCs, cell adhesion decreased since Day 4 (D4) as they underwent hematopoietic differentiation, resulting in suspension cells (Fig. 1b). These cells showed characteristics similar to those of HSPCs found in the AGM and the yolk sac. The cell differentiation process is shown in the flow cytometry (FCM) diagram (Fig. 1b). The proportion of CD44 (indicating naïve progenitors) [29, 30] was 99.85% on Day 2 (D2), which suggested that most of the cells differentiated in the expected direction. On D4, 97.58% of the cells expressed KDR^+^. By Day 10 (D10), most cells (97.74%) expressed both CD34 and CD43 markers, which indicated that these cells possessed some hematopoietic characteristics. On Day 12 (D12), the expression of CD31, CD34, CD43, and CD44 of the cells decreased by different degrees (Figure S1), suggesting terminal hematopoietic differentiation from HPSCs. We further investigated the CD34^+^CD43^+^HPSC colony-forming units (CFUs) observed on D10. It was found that these colonies were able to differentiate into various hematopoietic lineages, including erythroid cells, macrophages, granulocytes, GM (granulocytes and macrophages), and GEMM (granulocytes, erythrocytes, macrophages, and megakaryocytes) (Fig. 1c).

### Single-cell profiling during iPSC-to-HSPC process

To analyze the in vitro hematopoietic differentiation model, we collected the cells at six different time points: D0, D2, D4, D6, D8, and D10. After removing dead cells using the Dead Cell Removal Kit (Miltenyi), several analyses were performed on the collected samples, including scRNA-seq, DynaSCOPE, and ProMoSCOPE. The samples underwent multiple quality control and filtration steps before analyzation. We obtained high-quality transcriptome profiles of 24,584, 27,616, 32,402, 25,189, 30,185, and 28,500 cells respectively. Whole transcriptomes (total RNA) were obtained by scRNA-seq, newly synthesized transcriptomes (new RNA) were obtained by DynaSCOPE, and the glycosylation level of the cells was obtained by ProMoSCOPE [31].

We first selected D0, D4, and D8 to analyze the change in overall gene expression levels and obtained nine different expression patterns (Figure S2a). The results of GO enrichment showed that patterns 2 and 7 were associated with hematopoietic differentiation and regulation, and patterns 3 and 5 were involved in germ development (Figure S2b). In particular, the genes in pattern 1 were only highly expressed on D0 and D8 and were mainly involved in energy metabolism and protein translation, indicating that the middle stage of differentiation (D4) belonged to a state of low energy metabolism and low cell activity (Figure S2b).

**Fig. 2:**
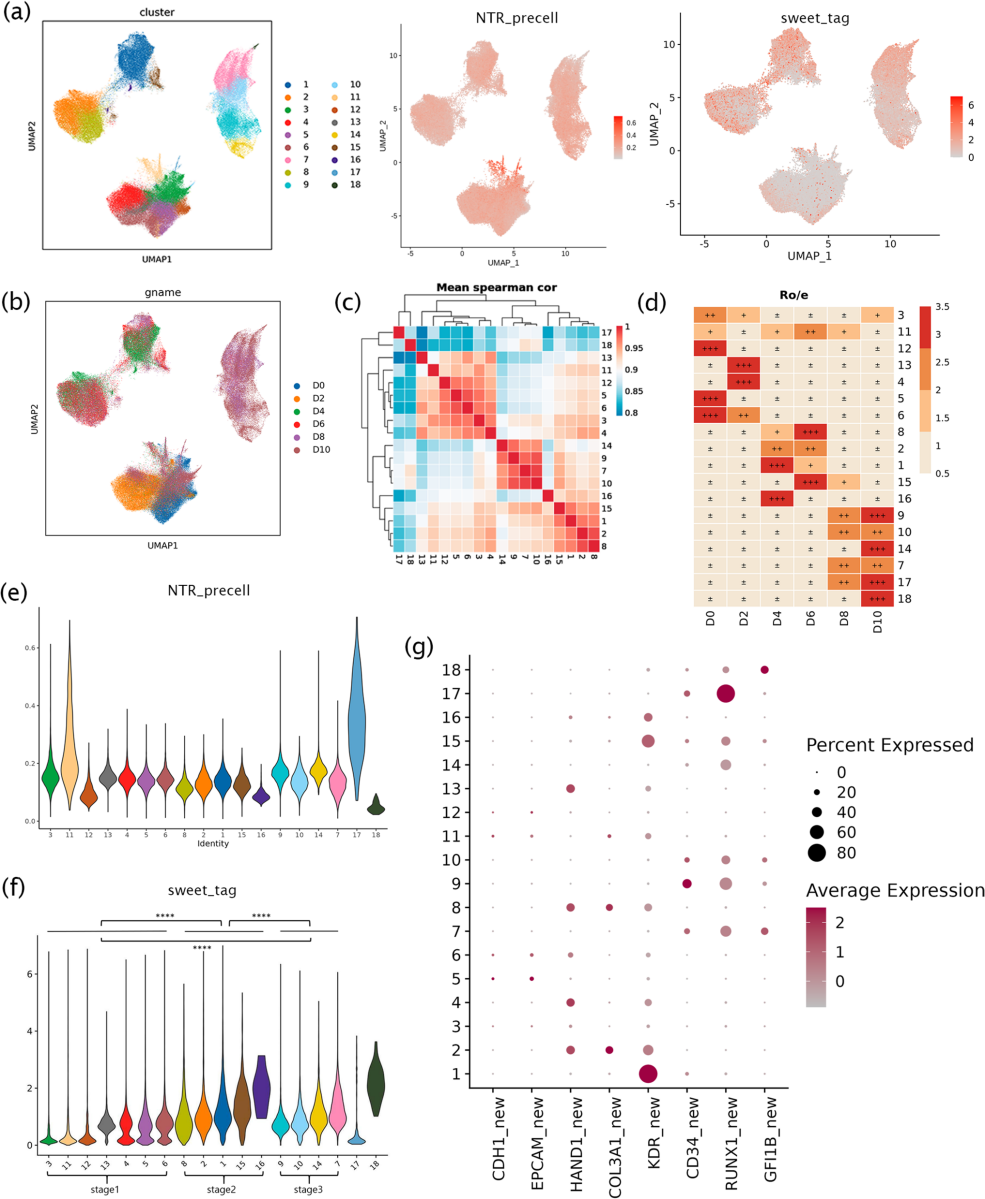
Single-cell profiling of iPSC-to-HSPC processes. **a**. Single-cell transcriptome, dynamic transcriptome, and glycosylation profiles of D0, D2, D4, D6, D8, and D10 samples were obtained during the iPSC-to-HSPC process, and clustered into 18 clusters. **b**. The UMAP plot of clusters from D0, D2, D4, D6, D8, and D10 samples. **c**. The Spearman correlation coefficient was calculated to determine the correlation between cell clusters and showed three stages of differentiation. **d**. The proportion of cells in a single sample with different clusters. **e**. The NTR of the dynamic transcriptome in 18 clusters. **f**. The level of glycosylation in 18 clusters. **g**. The expression of characteristic genes in different clusters in new RNA

The total transcriptome sequencing results identified 18 clusters based on gene expression, and the dynamic transcriptome and glycosylation profiles of the cells were shown based on the 18 clusters (Fig. 2a). We found that clustering occurred between samples (Fig. 2b and d) and the differentiation process could be divided into three main stages, based on the Spearman correlation coefficient between cell clusters (Fig. 2c). We classified D0 and D2 (clusters 3, 4, 5, 6, 11, 12, and 13) as stage 1 when iPSCs developed into the primary germ layer. D4 and D6 were classified as stage 2 (clusters 1, 2, 8, 15, and 16) and mainly included MCs and endothelial cells. Stage 3 included D8 and D10 (clusters 7, 9, 10, and 14), which mainly consisted of hematopoiesis-related cells. The new‐to‐total RNA ratio (NTR) reflects the degree of new RNA synthesis [31]. The dynamic data showed that the NTR was in the order of stage 3 > stage 1 > stage 2, with clusters 11 and 17 being the highest and clusters 18, 16, and 12 being the lowest (Fig. 2e). By the glycosylation at the LacNac site on the cell surface, it showed that the level of glycosylation was in the order of stage 2 > stage 3 > stage 1 (Fig. 2f). Similar to the results of the pattern of gene expression, with the results that the NTR was low, whereas the glycation level was high at stage 2, it was assumed that the cells were in a state of differentiation rather than proliferation at stage 2.

Through the expression of new RNA, we found that the main clusters of D0 (clusters 3, 5, 6, 11, and 12) in stage 1 highly expressed EPCAM (essential for the maintenance of the embryonic stem cell phenotype) [32], which indicated that they were still in the pluripotent stage. In addition, we found that the level of L1 TD1 [33], a downstream target of Nanog (a pluripotency marker), decreased as hematopoiesis progressed (Figure S2c). The main clusters of D2 (clusters 4 and 13) started to express the MCs markers HAND1 and COL3 A1, which indicated that D2 was in the stage of pluripotent cells developing to the primary germ layer. The early part of stage 2 (clusters 2 and 8) was associated with mesoderm development, but the high expression of KDR and CD34 in the late stage (clusters 1, 15, and 16) indicated that the cells entered hematopoietic differentiation. High expression of hematopoietic markers RUNX1 and GFI1B occurred in stage 3 (Fig. 2g). However, we found that clusters 17 and 18 were weakly correlated with others and were with highly expressed RUNX1 and GFI1B, respectively. The results of a GO analysis also showed pathways such as platelet activation in cluster 18, suggesting that these clusters may represent non-EHT differentiated hematopoietic cells.

### DynaSCOPE reveals the differentiation of cell clusters on pseudo-time

**Fig. 3:**
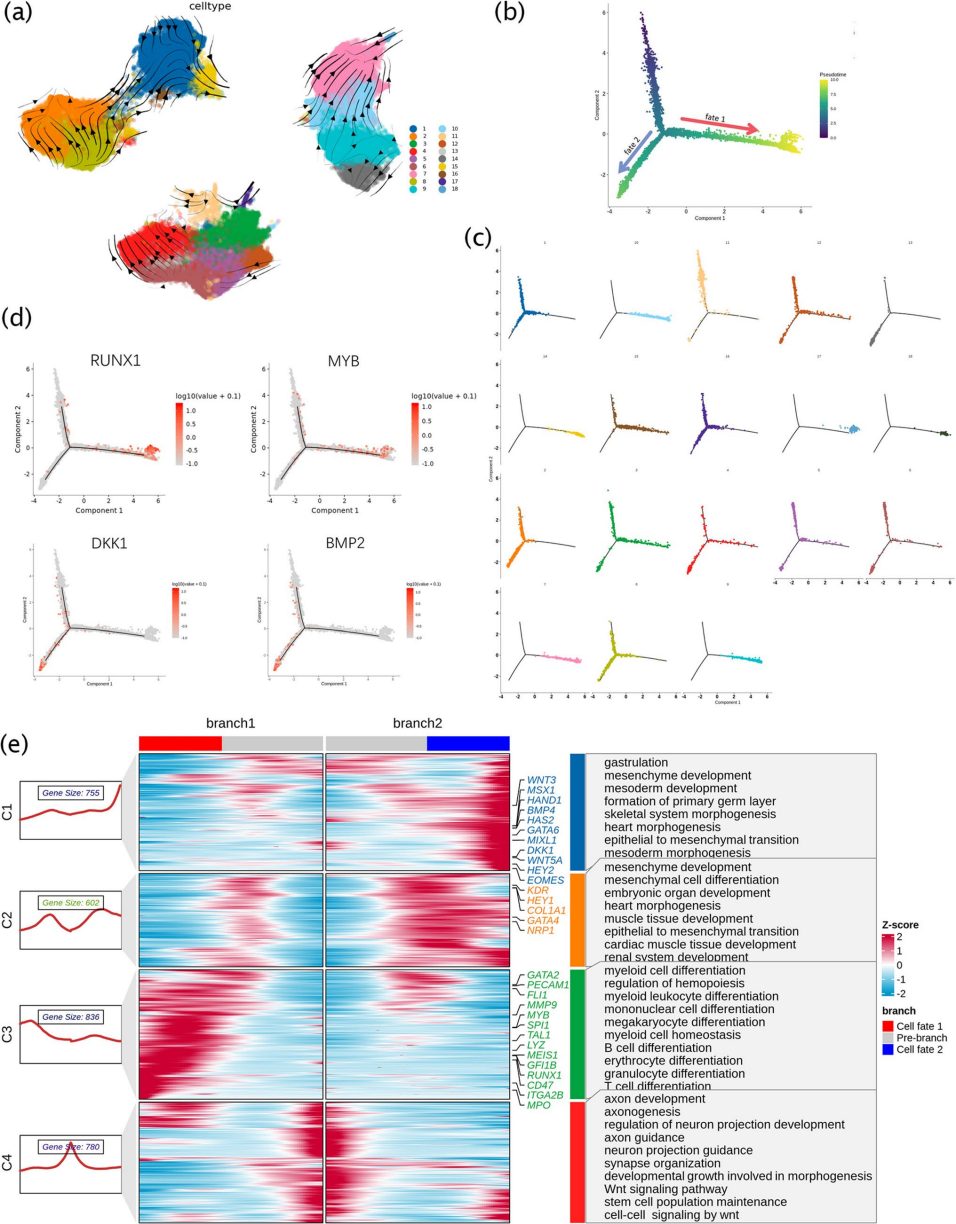
The differentiation of cell clusters on pseudo-time. **a**. RNA velocity was used to infer the differentiation trajectories and was indicated by the overlapping arrows in the UMAP plot. **b–c**. Monocle 2 analysis showed that the temporal variation at (**b**) and the clusters formation plot at (**c**), and the differentiation process was divided into two fates, the red arrow represented fate 1 and the blue one represented fate 2. **d**. The expression results of the genes were mapped on the pseudo-time. **e**. The BEAM branch was used to analyze the differential gene expression of two fate cells, and four clusters were formed

To study the fate of cell differentiation, we used Monocle2 to reconstruct single-cell trajectories in the complex differentiation processes. Due to the absence of FACS treatment during sample collection, different cell differentiation fates were detected, such as clusters 15 and 16 (Figure S3a and b, Fig. 3c). Therefore, we analyzed dynamic transcriptome data, focusing on the changes in new mRNA. The results of the RNA velocity analysis showed that the sequence of differentiation matched the sample time points and the inferred three stages of differentiation (Fig. 3a). The trajectory of clusters along the pseudo-time of differentiation can be divided into two differentiation fates (Fig. 3b and c). At pseudo-time (5), the distinct differentiation branches fate 1 and fate 2 appeared. The hematopoiesis regulation-related genes RUNX1 and MYB indicated that the hematopoietic branch was fate 1, while MIXL1 and DKK1 indicated that fate 2 was a mesoderm and endothelium-related lineage branch (Fig. 3d).

To further analyze gene expression for the two differentiation fates, we conducted a BEAM branch analysis of the new mRNA and obtained four clusters with similar status (Fig. 3e). The C1 gene was highly expressed at the endpoint of fate2, suggesting that it was developing toward the germ layer and embryonic organs (such as heart, muscle tissue, and renal system). The C2 gene enrichment showed that it was involved in germ development (such as gastrulation and the development of the mesoderm and mesenchyme) in the intermediate process in the direction of fate 1 and fate 2, indicating that hiPSCs had started to differentiate into potential or non-potential hematopoiesis cells. The biological processes enriched in C3 included hematopoietic regulation and differentiation of hematopoietic cells with different fates, suggesting that fate 1 is related to hematopoietic fate and function, and might result in erythroid lineage, myeloid lineage, and lymphoid lineage differentiation. The C4 gene was found to be involved in biological processes, such as axonal development, nervous system development, the mesoderm signaling pathway (Wnt signaling pathway), and stem cell population maintenance, indicating that some hiPSCs were still in the pluripotent stage, while others entered the differentiation process.

### Glycosylation and dynamic transcriptome analysis for the heterogeneity of endothelial clusters

**Fig. 4:**
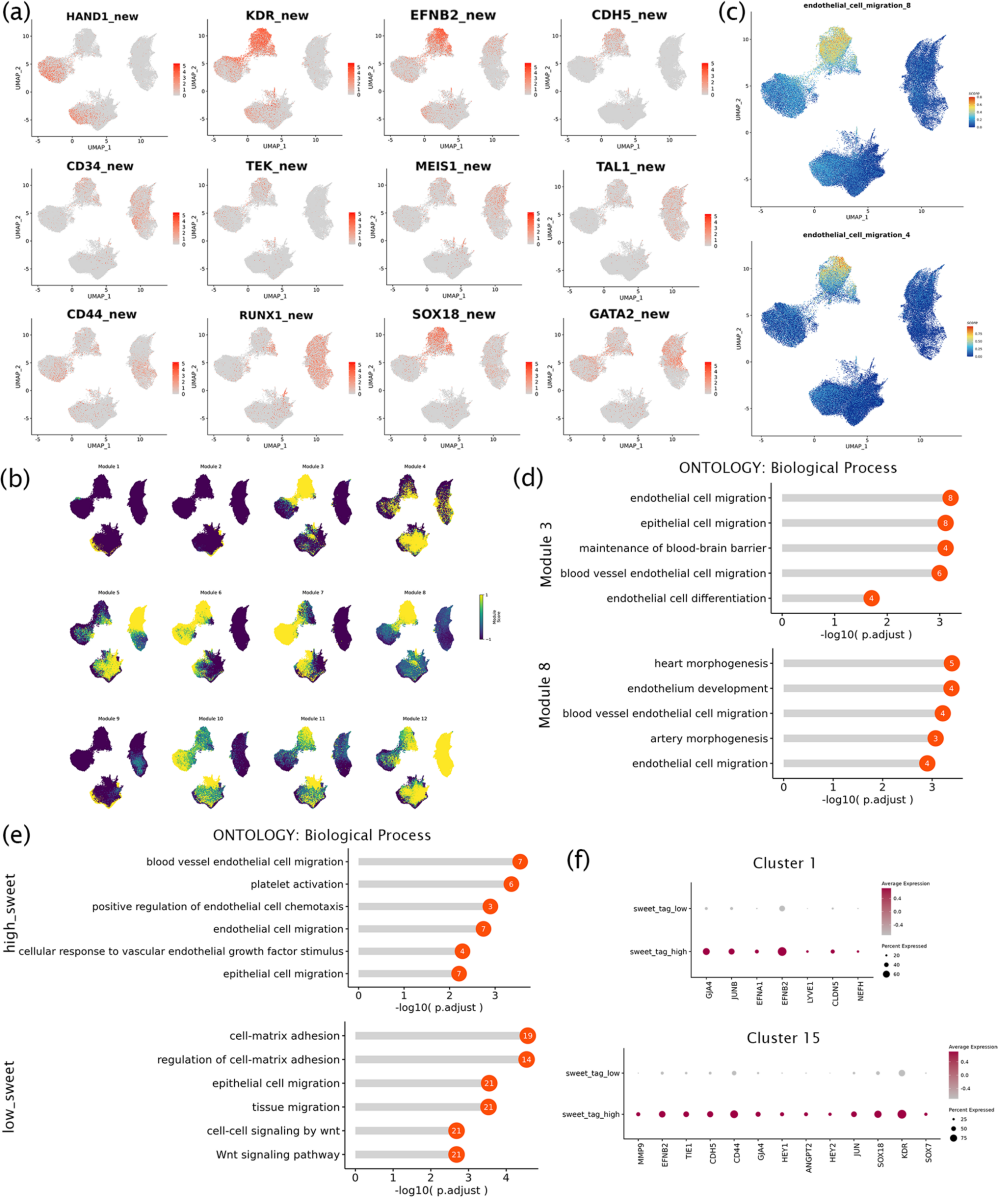
The analysis for the heterogeneity of endothelial clusters. **a**. The gene expression of endothelial, angiogenesis, and hematopoiesis in new RNA. **b**. The hotspot analyzed the similarity of cells and obtained 12 modules. **c–d**. The vascular endothelial cell migration pathway was selected from modules 3 and 8 and mapped to UMAP plots. **e**. The GO analysis of differential genes in high-glucose and low-glucose cells. **f**. The endothelial and hematopoietic genes in high-glucose and low-glucose cells in clusters 1 and 15

The results of the previous analysis showed that complex cell types occurred in stage 2. The lateral mesoderm marker HAND1 was expressed in stage 1. Early endothelial marker KDR and mature endothelial markers EFNB2 and CDH5 [34] were highly expressed in late stage 2. The different expression levels of hematopoietic marker CD34, vascular endothelial regulators TEK [35], MEIS1, and TAL1, hematopoietic naïve cell marker CD44 [29], and genes required for EHT processes (such as SOX18, RUNX1, and GATA2 [11, 36, 37]) at different stages suggested that EHT may begin in late stage 2 (clusters 1 and 15) (Fig. 4a). To further analyze vascular endothelial formation, 12 modules were obtained by hotspot analysis (Fig. 4b). The pathways for vascular endothelial cell migration were enriched in modules 3 and 8 (Fig. 4c and d), suggesting that HEC generation might occur in late stage 2 when the cells had complex endothelial subsets with high heterogeneity.

To investigate the significance of the level of glycation, we classified cells into high-glycemic cells (top 20% glycation abundance) and low-glycemic cells (bottom 20% glycation abundance). We found that high-glycemic cells were primarily enriched in vascular endothelial migration and platelet activation (Fig. 4E), which indicated that protein glycation plays a regulatory role in the generation of vascular endothelial cells [38]. Low-glycemic cells were mainly enriched in cell–matrix adhesion and the Wnt signaling pathway (Fig. 4e). These findings suggested that glycation levels may be related to cell viability and pluripotency. Through the high glycation level of stage 2 and the high expression of endothelial hematopoietic genes, we found that the expression of endothelial genes KDR, CLDN5, and CDH5, hematopoietic genes SOX18 and TIE1, and arterialization gene GJA4 increased in high-glycemic cells in clusters 1 and 15 (Fig. 4f), indicating that the development of the vascular system and the maturation of HECs were accompanied by glycation.

### Potential linkages of endothelial cell subsets in their differentiation fate

**Fig. 5:**
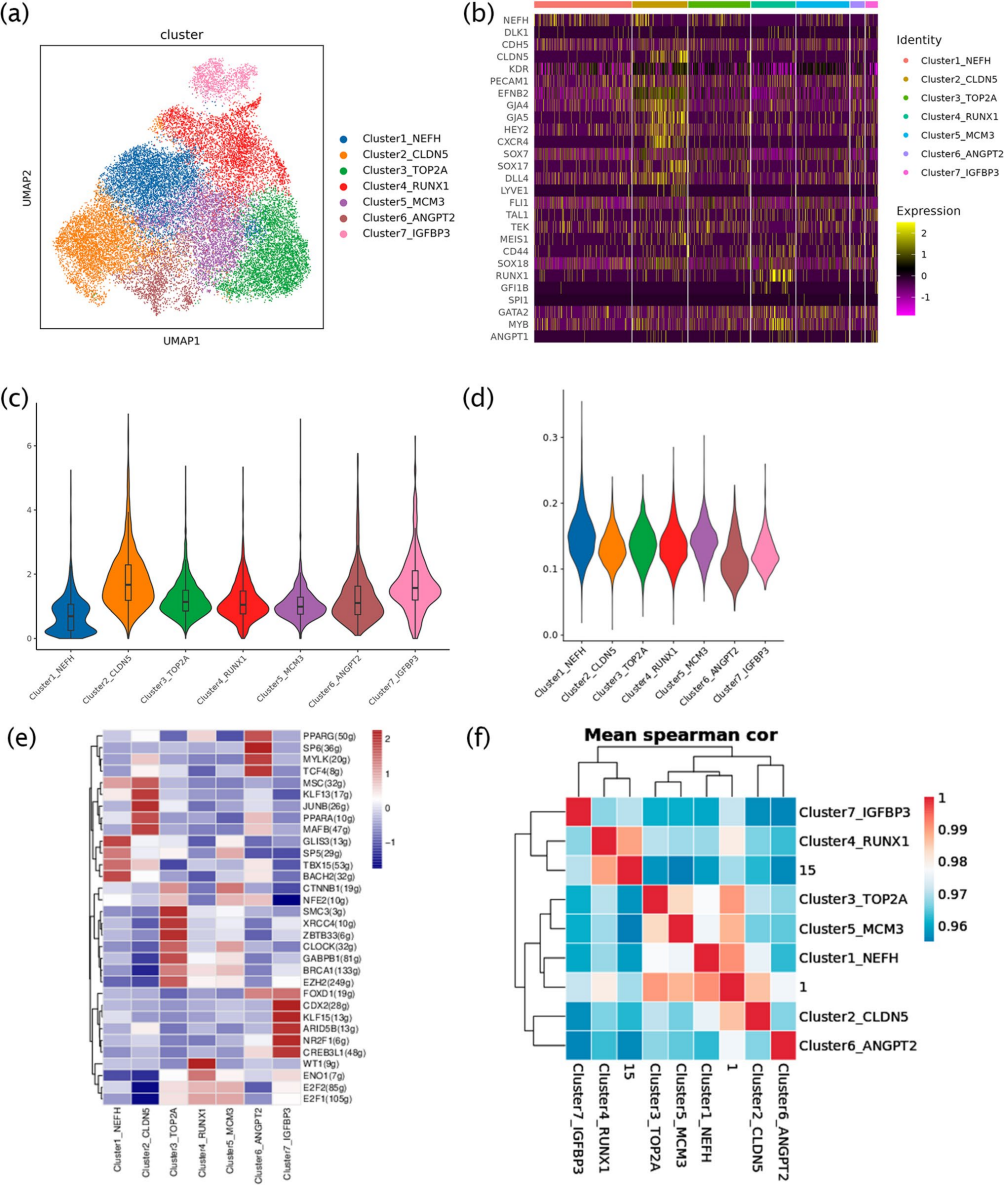
Potential linkages of endothelial subsets in their differentiation fate. **a**. Clusters 1 and 15 were reclustered to seven new clusters, which were identified by differentially expressed genes, including NEFH, CLDN5, TOP2 A, RUNX1, MCM3, ANGPT2, and IGFBP3. **b**. The expression of endothelial and hematopoietic-related genes in endothelial subsets. **c–d**. The NTR and the levels of glycation in endothelial subsets. **e**. The expression of transcription factors in the endothelial subsets. **f**. The correlation between clusters 1, 15, and the endothelial subsets using the Spearman correlation analysis

In vivo, HSCs are generated from HECs at the base of the dorsal aorta through EHT. HECs in blood vessels and other endothelial cells are derived from the same endothelial cell precursors [39]. We selected clusters 1 and 15 and reclustered seven clusters, which were identified as NEFH, CLDN5, TOP2 A, RUNX1, MCM3, ANGPT2, and IGFBP3, according to the most expressed genes of each cluster (Fig. 5a). It has been reported that lymphopoiesis requires the activation of the arterial program in HECs, while HECs without arterialization exhibit only primitive and myeloid-restricted hematopoietic potential [35, 40]. Hematopoiesis-related genes, such as RUNX1, CD44, GFI1B, SPI1, GATA2, and MYB, were highly expressed in cluster4_RUNX1, whereas endothelial genes (CDH5 and EFNB2) and angiogenesis regulators (TEK and FLI1) showed low expression in cluster4_RUNX1 (Fig. 5b), indicating that the cells lost endothelial characteristics and became precursor hematopoietic cells (pre-HE). Cluster2_CLDN5 included highly expressed arterial characteristic genes (GJA4, GJA5 [41], HEY2 [42], and CXCR4) and angiogenesis regulatory genes (DLL4 [43] and TEK). However, only SOX18 [36] and MEIS1 were highly expressed among hematopoietic regulatory genes in arterial endothelial cells (AECs) (Fig. 5b). Additionally, the expression of LYVE1 [44], a specific HSPC marker of vascular endothelial hematopoiesis in the yolk sac, was significantly upregulated in cluster2_CLDN5, suggesting that EHT of the yolk sac vessel might occur even in the absence of significant embryonic development in the in vitro hematopoietic model. JUNB can regulate EHT by binding to the promoters of key hematopoietic regulators or by promoting the deposition of hematopoietic transcription factors [45]. JUNB is highly expressed only in cluster2_CLDN5, which can help maintain the hematopoietic environment. Under such circumstances, the degree of glycation in endothelial subsets was opposite to that recorded for NTR (Fig. 5c and d).

During the development of embryonic hematopoiesis in vivo, HECs at different stages showed different differentiation fates [35]. Complex temporal variations in endothelial subsets (Figure S4) suggested that pre-HE had a weaker source relationship with AEC. The results of a correlation analysis between Clusters 1, 15, and the endothelial subsets confirmed that the fate of endothelial cells was determined early (Fig. 5f).

### The hematopoietic fate of iPSC-derived HSPCs

**Fig. 6:**
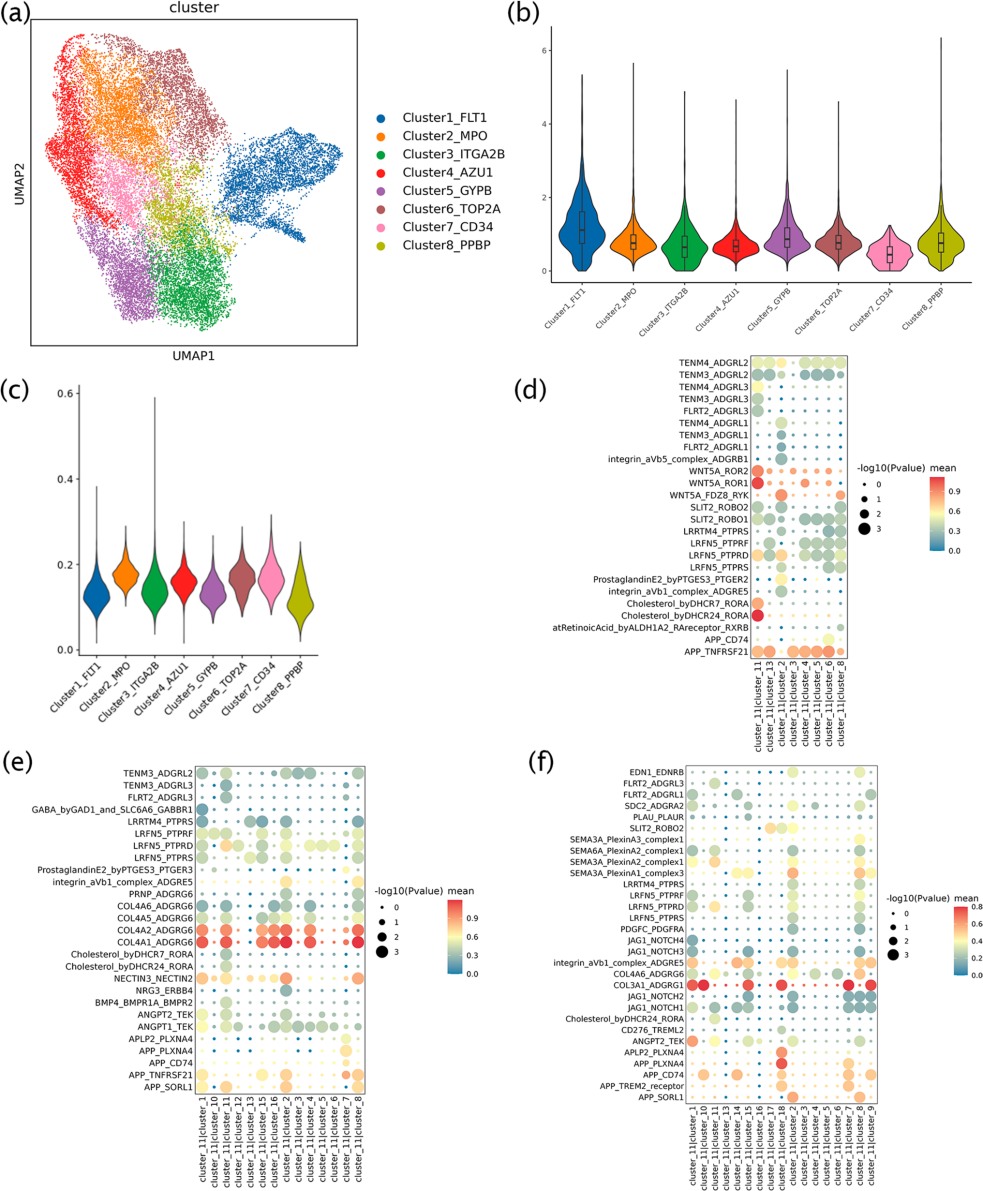
The hematopoietic fate of HSPC and the cellular communication with non-potential hematopoietic subsets. **a**. Clusters 9, 10, and 15 were reclustered to eight different clusters and identified by differentially expressed genes, including FLT1, MPO, ITGA2B, AZU1, GYPB, TOP2 A, CD34, and PPBP. **b–c**. The NTR and the levels of glycation in hematopoietic subsets. **d–f**. The bubble plots of the ligand–receptor pairs of cluster 11 and other clusters on D2, D4, and D8

The results of the similarity analysis between clusters 1, 15, and the endothelial subsets showed different developmental directions in the early development of endothelial subsets, with pre-HE mainly derived from cluster 15 and AECs derived from cluster 1 (Fig. 5f). In the hematopoietic differentiation at stage 3, cluster 7 was generated from cluster 9 and cluster 14 was generated from cluster 10, which indicated the emergence of hematopoietic progenitor cells with different fates. To analyze the relationship between the hematopoietic and endothelial cells, clusters 9, 10, and 15 were reclustered (Fig. 6a), and the eight resulting clusters were identified by the most expressed genes FLT1, MPO, ITGA2B, AZU1, GYPB, TOP2 A, CD34, and PPBP in each cluster, respectively. Two hematopoietic differentiation fates were identified in the results of the RNA velocity analysis (Figure S5a and b). Similar to the development of embryonic hematopoiesis in vivo, early megakaryocyte and erythroid cells were formed preferentially [35]. After entering hematopoietic differentiation, in most cells, the expression of cell-cycle pathway-related genes was high, which indicated that most of the obtained HSPCs differentiated continuously rather than existing as quiescent HSPCs. However, maintaining highly active HSPCs in an in vitro hematopoietic model is difficult and needs to be solved.

The analysis of glycation levels of hematopoiesis-related cells showed that the endothelial cluster1_FLT1 had the highest level of glycation, which was associated with the glycation of angiogenesis (Fig. 6b). Cluster7_CD34 with the lowest level of glycation (Fig. 6b), expressing ADGRG1 (marker of functional HSCs under oxidative stress during ex vivo culturing) [46] and ZFP36L2 (a critical modulator of definitive hematopoiesis) [47, 48], was speculated to be a transient subset of HSPCs. In addition, there was an opposite trend between the level of cellular glycosylation and NTR in these clusters. (Fig. 6b and c).

### Cellular communication between potential and non-potential hematopoietic cells

In the cellular communication analysis, we found that some non-potential hematopoietic subsets were still active in the late stage of differentiation. It has been showed that several types of stromal cell populations have different effects on hematopoietic development in vivo [35]. According to the characteristic genes in cluster 11, it mainly includes MCs and mesenchymal cells involved in differentiation and development. By comparing the differential genes of D0 and D8 in cluster 11, we found that the pluripotency genes were expressed on D0, whereas, COL3 A1, NRP1, HAPLN1, and TGFB1, which contributed to the maintenance of HSCs, were mainly expressed on D8 (Figure S5b). Therefore, we investigated the interactions between potential endothelial and hematopoietic subsets and cluster 11. At different stages of differentiation, cluster 11 communicated with potential endothelial and hematopoietic subsets. Cluster 11 maintained high activity throughout the differentiation process, which was mainly found in the expression of heterologous ligand–receptor for cell–cell interactions associated with the development and maintenance of hematopoietic signals. At different stages of differentiation, cluster 11 plays different roles. At stage 1, it acts as a ligand, interacting with ligand–receptors associated with the development of MCs and vascular endothelial growth (such as WNT5 A_ROR2 and VEGFB_FLT1) (Fig. 6d). At stage 2, it engages in ligand–receptor interactions associated with vascular endothelial growth (such as COL4 A1_ADGRG6) (Fig. 6e). At stage 3, cluster 11 acts as a ligand for hematopoiesis-related subsets cultured in vitro (such as clusters 7, 9, 10, 14, and 18) and also plays a role in regulation of oxidative stress and maintenance of cell self-renewal (such as COL3 A1_ADGRG1) (Fig. 6f) [46].

## Discussion

In this study, we used an in vitro hematopoietic differentiation model to construct a dynamic transcriptome profile and cellular glycosylation level of cells during iPSCs-to-HSPCs. Through the analysis of NTR and cellular glycosylation levels and the pseudo-time, it was revealed sequential stages of hematopoietic differentiation from iPSCs. On the hematopoietic differentiation axis, the expressions of MEIS1, GFI1B, RUNX1, MYB, and TAL1 were observed, which successively regulated hematopoietic events. Based on the heterogeneity of endothelial cells, it was found that HEC was more closely related to VECs than HSPCs, suggesting that the silencing of endothelial genes had a greater effect on HECs.

The degree of glycation was significantly different at different stages of hematopoietic differentiation (Fig. 2f). The high-glycemic cells in the endothelial subsets of stage 2 enriched angiogenesis process and the hematopoietic subsets in stage 3 enriched activation of platelets. As cells mature, the level of glycosylation gradually increases. However, we found that the level of cellular glycosylation in endothelial subsets was higher than in hematopoietic subsets, especially in clusters 1 and 15 in stage 2. In other studies, microRNA-223 was found to be ubiquitous in HECs and nascent HSPCs, and it limited EHT in lympho-myeloid cells by inhibiting the expression of the mannosyltransferase alg2 and sialyltransferase st3 gal2 (two glycosylation regulators) [17]. By comparing the differential genes of high and low-glycemic cells, we identified many genes involved in hematopoietic regulation while associated with endothelial cells (Fig. 4f), indicating that there was a unique glycation mechanism regulating the EHT. In the hyperglycemic cells in stage 3, the overall glycation level of the granulocyte–macrophage progenitor cells was lower than that of the megakaryocytic–erythroid progenitor cells. Moreover, it was found that megakaryocytic–erythroid progenitor cells were mainly enriched in the biological processes of platelet activity, indicating that high platelet activity may be related to high glycation. In addition, other biological regulatory processes may mediate hematopoietic development through protein glycosylation, although the precise mechanisms require further investigation.

Some differentiation events in the iPSCs-to-HSPCs model recapitulate key aspects of in vivo hematopoietic development. During embryonic hematopoietic development, definitive hematopoiesis begins with HECs, which are generated from the dorsal aorta in AGM. Consequently, the development of hematopoietic endothelial subsets is intrinsically linked to arterial development. Notably, a unique group of HECs has been reported in early human embryos that lack arterial characteristics and express a tendency toward macrophage differentiation [35]. In differentiation models, arterial gene expression may affect the comprehensiveness of hematopoietic differentiation, especially in lymphatic fate. In endothelial subsets, cluster2_CLDN5 and cluster4_RUNX1 have different expression of arterial genes (GJA4 and GJA5 [41]) (Fig. 5b), suggesting a unique HECs in cluster4_RUNX1. Upregulated genes in stage 3 were associated with the cell cycle and DNA replication, similar to the cycling HSPC subsets present during CS13 and CS15 in vivo [35]. Thus, promoting arterial endothelial gene expression and cell-cycle regulation may increase the potential of HECs differentiating into HSCs in vitro. Based on these results, it may be concluded that the model in vitro follows a similar process to that observed in vivo.

During embryonic hematopoiesis, various epithelial cells and MCs assist in hematopoiesis near the CS15 dorsal aorta, but the specific molecular regulatory roles are unclear. It was found that cluster 11 had information transmission with potential endothelial and hematopoietic subsets at different stages, indicating that there was a process to maintain the hematopoietic environment by differentiating stromal cells. During hematopoietic development in vivo, it was found that stromal cells, including mesenchymal and epithelial cells, were present in the AGM region, to assist in maintaining hematopoiesis. Additionally, clusters 17 and 18, which were weakly associated with all three stages and had high hematopoietic genes, were possibly associated with hematopoietic cells generated from HSCs-independent EHT.

**Fig. 7:**
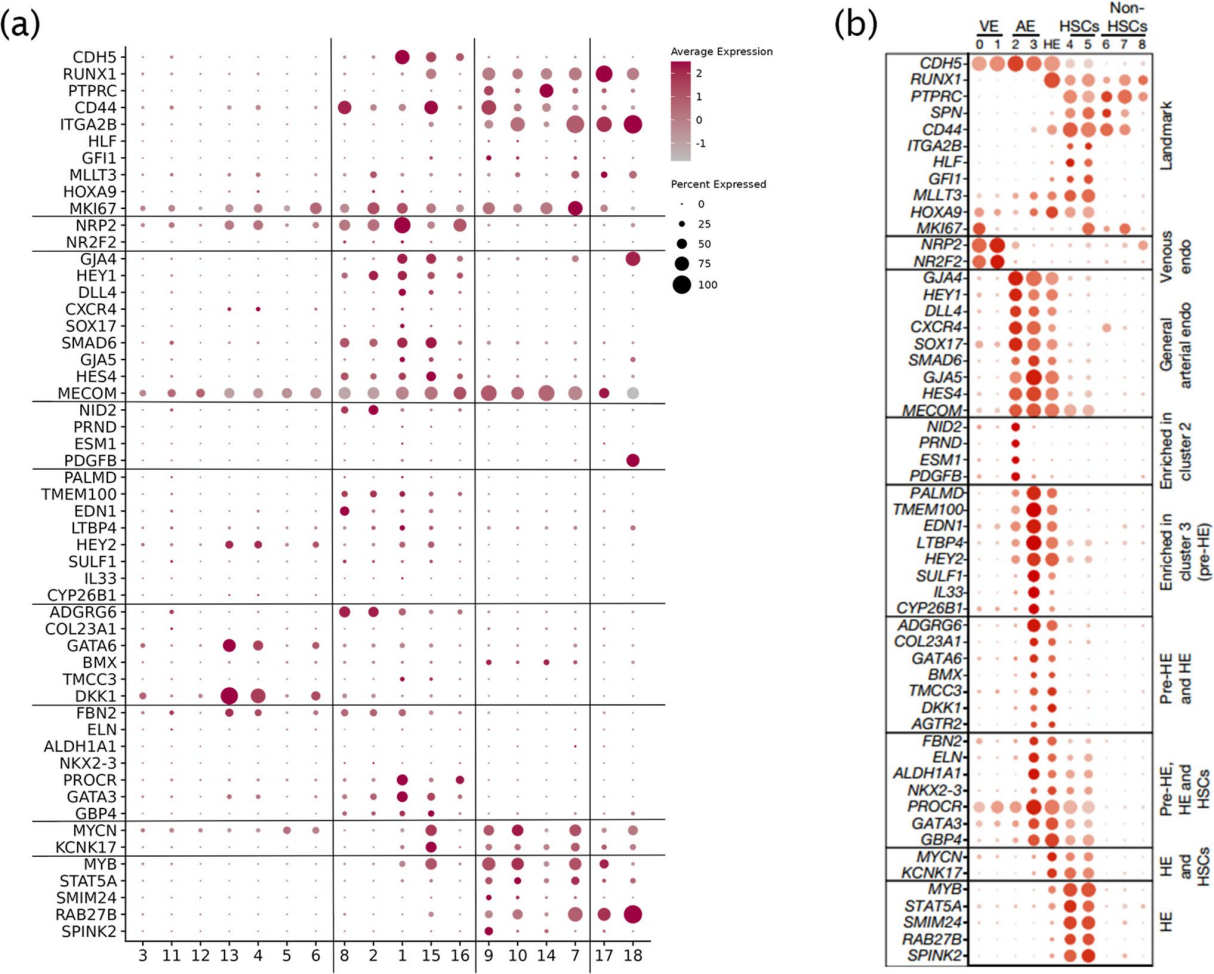
EHT transformation-specific gene scorecard [49]. **a–b**. The differentially expressed genes of the differentiation process in hematopoietic model in vitro were shown in (**a**), and the differentially expressed genes of embryonic hematopoiesis in CS14 and CS15 in vivo were shown in (**b**) [49]

The EHT scorecard (Fig. 7a and b) was used to demonstrate the expression of cell clusters at different stages, showing similarities between endothelial hematopoietic waves (pre-HE, HECs, and HSCs) in vivo and hematopoietic differentiation in vitro [49]. GATA6 and DDK1 are important transcription factors and markers for liver development [50], which have been fully expressed in stage 1 during fetal organ development, whereas IL33 and ALDH1 A1, the specific markers of the endothelial precursors that generate HSCs in vivo, have not been found in vitro, indicating that although there are many similarities between the in vitro model and in vivo hematopoiesis, there are still differences as it is difficult to reproduce the hematopoietic microenvironment in vitro.

While this study provides valuable insights, several limitations should be acknowledged. First, due to experimental constraints, we were unable to perform repeated differentiations using multiple independent cell lines. This limitation may impact the generalizability of our findings, as variations in genetic backgrounds or epigenetic states between cell lines could lead to differences in differentiation efficiency or functional outcomes. Consistent with established hematopoietic differentiation paradigms, this approach reliably produces multiple blood cell types through well-characterized progenitor cell lineages, as demonstrated in the previous studies [11, 14]. Nevertheless, our single-cell transcriptomic analysis of differentiated hematopoietic stem cells populations revealed conserved expression patterns of key hematopoietic regulators that were consistent with established gene expression patterns reported in the previous studies [35, 40, 41, 49]. A further challenge arises from the difficulty of obtaining human embryonic hematopoietic stem cells at multiple developmental stages simultaneously, which precluded experimental validation of our single-cell analysis findings using in vitro assays. Future studies could address these limitations by expanding the range of cell lines tested or employing animal models to further validate the conclusions drawn from this work.

## Conclusions

Overall, many differentiation events in the in vitro model are similar to hematopoietic development in vivo, including yolk sac hematopoiesis, cellular communication between non-potential hematopoietic subsets and potential hematopoietic subsets, gene expression, and temporal deviations in hematopoietic fate. Our study advances hematopoietic development understanding and HSC production for therapy, though limited by single-line derivation and lack of embryonic validation, requiring future multi-line and in vivo studies.

## Supplementary Information

Below is the link to the electronic supplementary material.

Supplementary file1 (DOCX 2318 KB)

Supplementary file2 (DOCX 17 KB)

## Data Availability

The datasets generated and analyzed during the current study are available in the Gene Expression Omnibus (GEO) repository with Accession Number: GSE263152, https://www.ncbi.nlm.nih.gov/geo/query/acc.cgi?acc=GSE263152.
