# Supplementary material for: Single-cell dynamic RNA and glycosylation sequencing reveals the mechanism underlying the differentiation of pluripotent stem cells into hematopoietic stem cells
Source: Hum Cell. 2025 May 27;38(4):110. doi: 10.1007/s13577-025-01234-7 (PMC12116958; doi:10.1007/s13577-025-01234-7)
Supplement: Supplementary file 1 — Supplementary file1 (DOCX 2318 KB) [file 13577_2025_1234_MOESM1_ESM.docx]

Supplementary Figure_S1


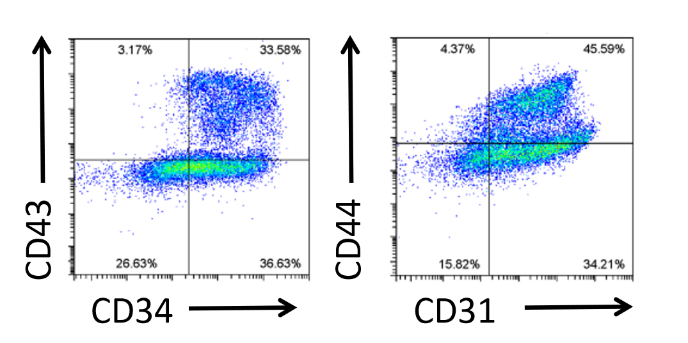


Figure_S1. The gene expression of hematopoietic markers in D12 samples.

Supplementary Figure_S2


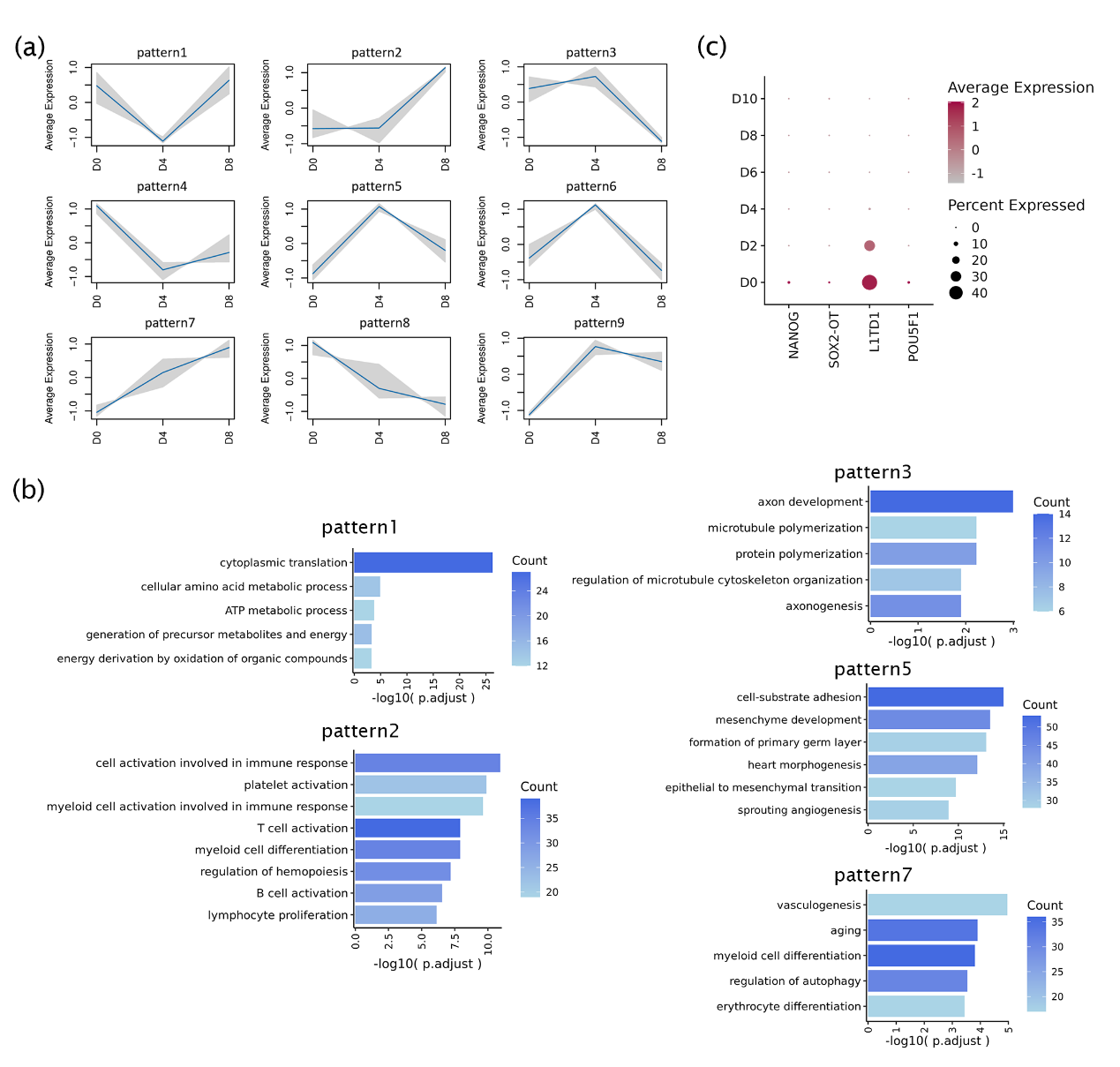


Figure_S2. The analysis of transcriptome gene expression patterns. (a). Nine gene expression patterns in the transcriptome were analyzed at three time points (D0, D4, and D8). (b). The gene enrichment pathways associated with patterns 1, 2, 3, 5, and 7. (c). The bubble plot of the expression of pluripotency genes in different samples.

Supplementary Figure_S3


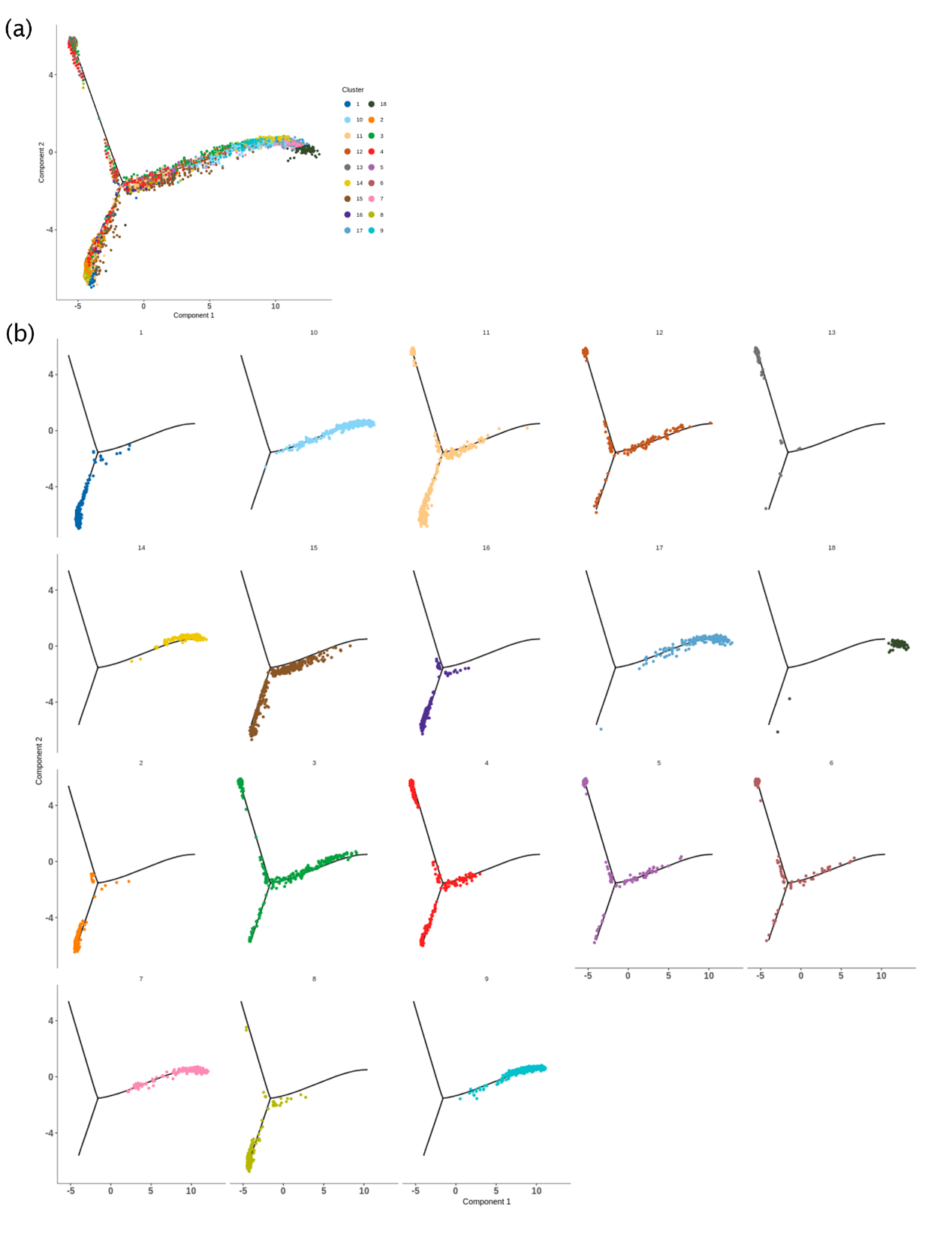


Figure_S3. The cluster distribution of the cells on pseudo-time. (a) is a merged result of (b).

Supplementary Figure_S4


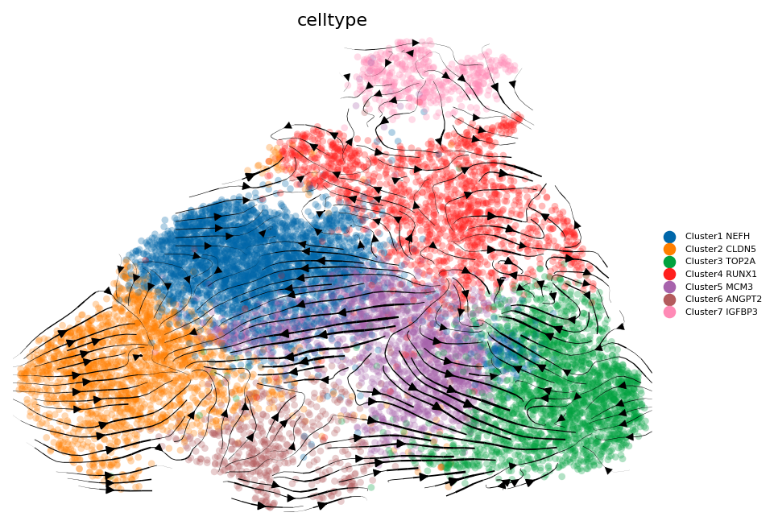


Figure_S4. The endothelial subsets differentiation trajectories.

Supplementary Figure_S5


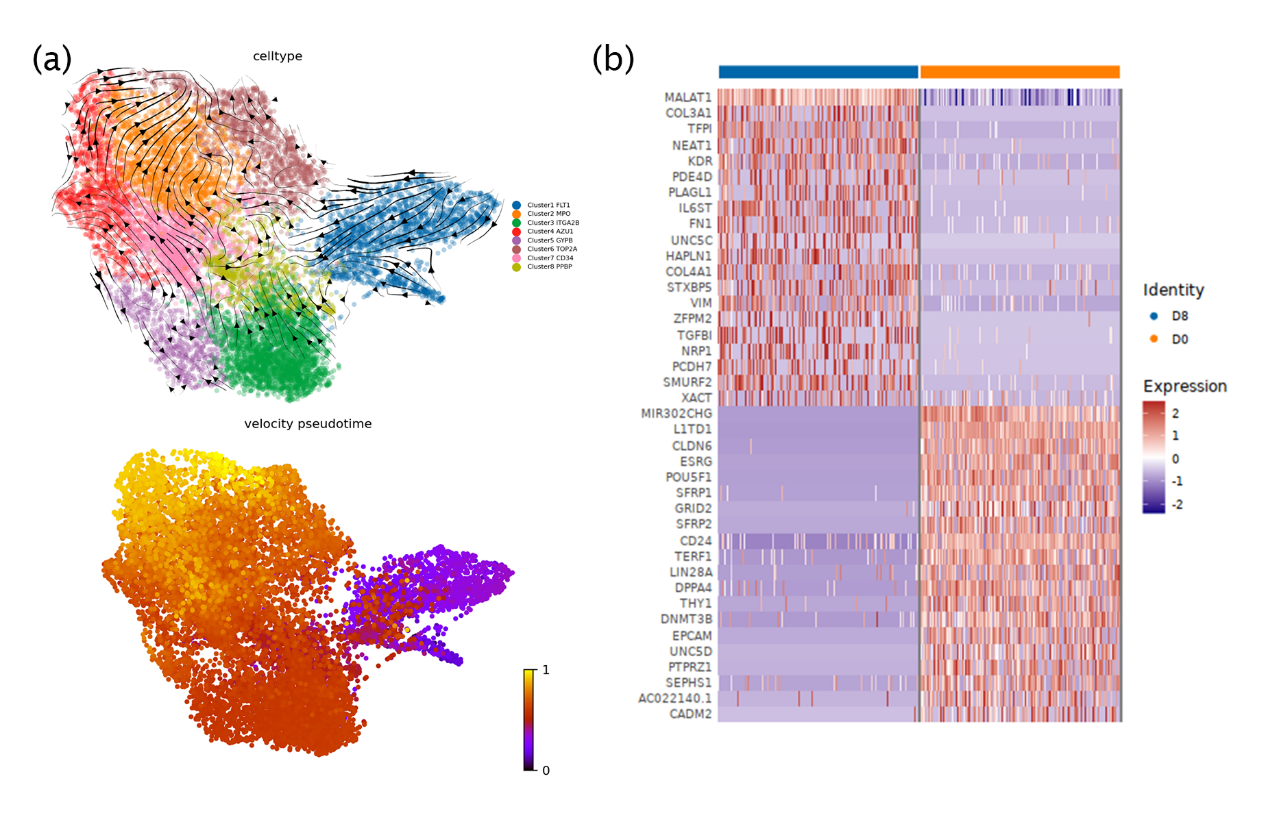


Figure_S5. The differentiation fate of hematopoietic cells. (a). The hematopoietic subsets differentiation trajectories. The arrows represented the differentiation fate of the cells, while the purple represented pseudo-time (0), and the yellow represented pseudo-time (1). (b). The differentially expressed genes on D0 and D8 in cluster 11.
