# Supplementary material for: Single-cell dynamic RNA and glycosylation sequencing reveals the mechanism underlying the differentiation of pluripotent stem cells into hematopoietic stem cells
Source: Hum Cell. 2025 May 27;38(4):110. doi: 10.1007/s13577-025-01234-7 (PMC12116958; doi:10.1007/s13577-025-01234-7)
Supplement: Supplementary file 2 — Supplementary file2 (DOCX 17 KB) [file 13577_2025_1234_MOESM2_ESM.docx]

Table 1 The antibodies were used for flow cytometry analysis and cell sorting.

| Antibodies | Cat. No. | Isotype control | Cat. No. | Company |
| --- | --- | --- | --- | --- |
| PE anti-human CD309 (VEGFR2) | 393003 | PE Mouse IgG1, κ Isotype Ctrl | 400111 | BiolegendClone |
| PE anti-human CD31 | 303105 |  |  | BiolegendClone |
| PE/Dazzle™ 594 anti-human CD184 (CXCR4) | 306525 | PE/Dazzle™ 594 Mouse IgG2a, κ Isotype Ctrl | 400275 | BiolegendClone |
| PE/Dazzle™ 594 anti-human CD34 | 343533 | PE/Dazzle™ 594 Mouse IgG1, κ Isotype Ctrl | 400177 | BiolegendClone |
| PE/Cyanine7 anti-human CD44 | 338815 | PE/Cyanine7 Mouse IgG1, κ Isotype Ctrl | 400125 | BiolegendClone |
| FITC Anti-human TRA-1-60 | 560876 | FITC Mouse IgM, κ Isotype Ctrl | 553474 | BD |
| APC anti-human SSEA-4 | 330417 | APC Mouse IgG1, κ Isotype Ctrl (FC) | 400121 | BiolegendClone |
| APC anti-human CD43 | 343205 |  |  | BiolegendClone |
| Zombie Aqua™ Fixable Viability Kit | 423101 | \ | \ | \ |
